# Beyond the bamboo ceiling: How ethnic collaborative networks facilitate Chinese inventors’ knowledge remittances

**DOI:** 10.1371/journal.pone.0347548

**Published:** 2026-04-17

**Authors:** Hua Zhang, Jia Liu, Chunhui Cao

**Affiliations:** 1 College of Business Administration, Huaqiao University, Quanzhou, China; 2 The School of International Business and Management, Shanghai International Studies University, Shanghai, China; University of Washington, UNITED STATES OF AMERICA

## Abstract

Previous research on the “bamboo ceiling” has focused on how ethnic homophily limits career advancement for East Asian immigrants. However, less is known about how their ethnic collaborative networks as U.S. Chinese immigrant inventors may facilitate knowledge remittances to their home countries. Drawing on social network theory, this study investigates two core network positions—centrality and structural holes (i.e., connecting otherwise unconnected co-inventors)—and their effects on knowledge remittances. Ethnic knowledge utilization serves as the mediator, while inventors’ past performance acts as the moderator. We test the proposed relationships using a unique dataset of patents in the integrated circuit industry. Results show that ethnic knowledge utilization mediates the relationship between network positions (centrality and structural holes) and knowledge remittance. Moreover, past performance significantly moderates the mediating paths: high-performing inventors more effectively exploit structural holes to facilitate knowledge remittance. They also strengthen the curvilinear effect of centrality, accelerating knowledge remittance at optimal centrality levels but triggering an earlier decline beyond these thresholds due to stronger path dependency. These findings not only support policies for leveraging diaspora knowledge networks but also advance the literature by establishing a more nuanced, attribute-based micro-foundation for understanding high-skilled migration’s impact on home-country development.

## 1. Introduction

A central question in migration research is how the rising movement of migrants—particularly high-skilled migrants—fuels development in their home countries [1]. While knowledge remittances have attracted considerable attention, prior work has focused largely on macro-level dynamics, leaving the micro-level, individual-based mechanisms underpinning their generation remain underexplored [2,3]. Such mechanisms are inherently embedded in ethnic networks: prior research acknowledges East Asian migrants’ tendency toward ethnic clustering, yet it predominantly frames this phenomenon through the “bamboo ceiling” lens—a phenomenon describing the underrepresentation of East Asians in leadership positions, partly attributed to high ethnic homophily (i.e., a preference for within-group socialization) [4]. This perspective overlooks a critical alternative: robust ethnic collaborative networks may also serve as a key driver of knowledge remittances. This study therefore moves beyond the “bamboo ceiling” paradigm by positing that while strong intra-ethnic ties may constrain leadership advancement, they can simultaneously facilitate the legitimization and transnational flow of specialized knowledge—enabling skilled immigrants to contribute effectively to their home countries’ development through knowledge remittances. Consequently, a deeper understanding calls for a closer examination of how ethnic collaborative networks shape the generation of knowledge remittances.

We propose that ethnic collaborative networks serve two functions: they act as quality cues for home-country inventors evaluating immigrant inventors’ knowledge [5,6], and as conduits for accessing and recombining ethnic knowledge, which in turn raises the probability of home-country citations for patents enriched with ethno-specific insights [7]. Specifically, we argue that distinct network positions reflect different quality-relevant attributes: structural holes denote access to novel knowledge, while centrality implies credibility. These attributes influence both the perceived value of immigrant knowledge and its likelihood of domestic citation. At the same time, these networks enable immigrant inventors to access and apply ethnically embedded knowledge [8,9]. We further propose that an inventor’s past performance moderates this channel mechanism: inventors with higher past performance are better able to assimilate and leverage ethnic knowledge, thereby increasing knowledge remittance. Thus, this paper advances an integrated understanding of how immigrant inventors’ ethnic collaborative network functions as both quality indicators and transmission channels, and how past performance conditions the knowledge remittance process.

We test our hypotheses using a unique sample of Chinese immigrant inventors (hereafter “Chinese inventors”) in the United States, constructed through a specialized patent data screening approach. Our empirical analysis reveals three main findings. First, we observe an inverted U-shaped relationship between centrality in ethnic collaborative networks and knowledge remittance: moderate centrality promotes knowledge remittance while excessive centrality reduces it. In contrast, structural hole positions consistently facilitate knowledge remittance. Second, the effects of these networks on knowledge remittance is mediated by the extent of ethnic knowledge utilization. Third, we demonstrate that individual past performance significantly moderates these relationships: it amplifies the inverted U-shaped effect of network centrality on ethnic knowledge utilization, reinforces the positive impact of structural holes, and further moderates the indirect effect via ethnic knowledge utilization. Together, these results highlight how network structure and individual past performance collectively govern the dynamics of knowledge remittance.

This paper is structured as follows: Section 2 introduces the theoretical framework and proposes the research hypotheses; Section 3 delineates the research methodology, encompassing data sources and processing procedures, the construction of ethnic collaborative networks, the operationalization of key variables, and identification strategy with considerations for endogeneity; Section 4 presents the empirical analysis, employing regression models to test the hypotheses alongside robustness checks; finally, Section 5 synthesizes the conclusions, discusses the study’s theoretical contributions and limitations, and suggests directions for future research.

## 2. Related works and hypothesis

### 2.1 Migrant inventor, ethnic knowledge and ethnic collaboration

Rooted in shared cultural identity, values, and social trust, immigrants tend to form ethnic communities in host countries that not only strengthen group cohesion but also deliver economic benefits through resource sharing and information exchange [10,11]. High-skilled migrants’ ethnic collaborative networks represent a typical form of such communities. As Saxenian (2002) demonstrated, overseas engineers and scientists—perceiving themselves as marginalized in host societies—often establish strong ethnic ties to enable knowledge flows [12]. These communities function as conduits for transferring context-specific knowledge embedded in home-country cultural and institutional environments, which may otherwise remain “sticky” due to linguistic barriers, causal ambiguity, or unfamiliarity in host regions [13,14]. While ethnic clustering is common among global migrants, East Asian immigrants exhibit particularly strong tendencies due to collectivist cultural norms [4]. In the U.S. technology sector, Chinese inventors show distinct preferences for intra-ethnic collaboration, forming co-invention networks with a disproportionately high share of Chinese collaborators [15]. These preferences are reinforced by altruistic norms and enforceable trust within ethnic networks, which lower transaction costs and enhance the salience of community-sourced knowledge [16]. While these patterns highlight the functional advantages of ethnic networks, it is equally important to understand the underlying forces that shape their formation and evolution.

Building on these observations, we note that the formation of such networks is far from random; rather, it is endogenous, shaped by a confluence of cultural, institutional, and technical factors. A primary driver is homophily—the tendency for individuals to associate with similar others [17,18,19]. This manifests in ethnic networks as preferential attachment based on shared language, educational background, and professional identity, directly reinforcing the high intra-ethnic collaboration density observed among Chinese inventors [19]. However, the extent and nature of this insularity are not uniform. For instance, in the Integrated Circuit (IC) industry, the need to access specialized, tacit knowledge for components where China historically has had less solid expertise may push some co-ethnic networks to be more open to inter-ethnic collaboration, whereas other subfields may remain more closed—reflecting how contextual and technical demands can moderate the homophily-driven segmentation documented in prior network research [19,20]. This variation in openness, in turn, may influence how effectively these networks facilitate the transfer of different types of ethnic knowledge—a key contingency that shapes their ultimate role in driving knowledge remittances, as network structure and position have been shown to directly impact the efficiency of knowledge flow and resource transfer [21]. We draw on this literature to frame our analysis, positioning our work within the broader discourse on how social structures formation while focusing on how such ethnic network structures shape cross-border ethnic knowledge transfer and subsequent knowledge remittances.

Against this backdrop, immigrant inventors actively draw on ethnic knowledge for innovation, with their utilization shaped by both capability considerations and career incentives. From the capability perspective, their familiarity with home-country contexts enables efficient identification and utilization of ethnic knowledge [14]. Extended immersion in home-country knowledge traditions grants them absorptive capacity to decode tacit and complex knowledge embedded in local contexts [22]. Even when encountering unfamiliar ethnic knowledge, shared linguistic and cultural backgrounds significantly substantially reduce acquisition barriers [23]. From the career concern perspective, facing dual pressures to establish reputation and advance professionally in host countries [24], immigrant inventors show a marked preference for applying ethnic knowledge in incremental innovation, as reflected in disproportionately high citations to home-country patents. However, over-embeddedness in ethnic collaborative networks may trigger diminishing returns or even reduce innovation quality when inventors rely excessively on redundant community knowledge. This highlights an inverted U-shaped relationship between ethnic knowledge sourcing and innovation impact [8,25].

Within ethnic collaborative networks, shared cultural backgrounds and trust mechanisms reduce transmission costs for tacit and complex knowledge from home country [26]. We define ethnic knowledge as that embodied in patents filed by home-country inventors —a measurable construct that grounds our empirical analysis of knowledge remittances. Immigrant inventors primarily reuse such knowledge directly in host-country innovation, while ethnically mixed teams drive knowledge recombination by integrating it with other technological components [14]. Strong ethnic ties not only ensure reliable transfer but also establish reciprocal feedback loops that drive technological innovation. This collaborative pattern integrates technical perspectives from both origin and host countries, yielding diversified solutions for novel applications—thereby enhancing the scalability and transferability of ethnic knowledge for subsequent remittance. This recombination process is further amplified when such knowledge is transferred into host firms with robust institutional support, such as specialized services or strong IP regimes, which mitigates home-country institutional voids and boosts remittance effectiveness [27].

### 2.2 Ethnic collaborative network, ethnic knowledge utilization and knowledge remittances

Consistent with prior research [28,29], we define knowledge remittance as the process in which Chinese inventors’ patents are cited by Chinese patents, reflecting the flow of knowledge from overseas back to the home country. Operationally, this corresponds to home-country forward citations to Chinese inventors’ patents. While patent documents are public, the core challenge for home-country inventors lies not in access to this knowledge, but in efficiently identifying and evaluating which overseas knowledge holds the greatest potential for valuable recombination within their local contexts. This process is fraught with uncertainty due to geographic, cognitive, and institutional distances [30]. Drawing on the literature on knowledge search and recombination under uncertainty [5] as well as insights from Nee’s research on Chinese production networks [31], we posit that the structural positions of inventors within ethnic co-invention networks serve as critical heuristics. These positions help home-country inventors assess the potential value and applicability of distant knowledge, especially when institutional and contextual ambiguities exist in cross-border transfers. They act as credible indicators of knowledge quality, thereby reducing search and evaluation costs during technology screening.

Uzzi and Spiro (2005) provided a foundational argument for the importance of network structure in circulating creative materials [32]. They showed that small-world networks—characterized by a mix of local clustering and short global paths—enhance innovation by improving connectivity. Crucially, they found this relationship to be non-linear, with an inverted U-shape. While moderate connectivity optimizes the flow of ideas, excessive connectivity leads to information homogenization and stagnation. Building on this logic, we contend that in ethnic collaboration, specific structural positions—namely centrality and bridging positions (structural holes)—play distinct roles in shaping the likelihood of knowledge remittance.

We first posit that the number of structural holes an inventor spans is positively associated with the likelihood of their knowledge being cited by the home country. In this study, a structural-hole broker refers to a bridging position that connects co-ethnic inventors who would not otherwise collaborate directly, or that links otherwise isolated knowledge domains [33]. By occupying such a position, the broker gains access to non-redundant information from diverse, independent groups—information that would be unavailable within closed, insular networks—enhancing the novelty and recombinant potential of their innovations. Such a position offers two direct advantages for home-country evaluators. First, it provides access to non-redundant information from diverse knowledge streams that are unavailable in more insular clusters. Second, it requires the integration and translation of disparate knowledge, which often enhances the novelty and recombinant potential of the inventor’s own output [34].

From the perspective of a home-country inventor, the bridging position serves as a powerful inferential cue. It indicates that the knowledge originating from this node is more likely to be (a) novel, as it draws on unique combinations, and (b) generatively rich, as it has already undergone a synthesis process across boundaries. This makes such knowledge a highly attractive input for recombination, as it offers the possibility of creating something new and valuable that is not readily available elsewhere. Therefore, the brokerage signature is a positive indicator of the potential value of their knowledge, increasing its probability of being noticed, deemed relevant, and ultimately cited by home-country peers.

In contrast, we argue that an inventor’s degree centrality exhibits an inverted U-shaped relationship with the likelihood of being cited by the home country. At moderate levels, centrality is beneficial. A central position indicates deep embeddedness and engagement with a core group of peers [35]. This sustained interaction implies the inventor’s work is consistently vetted and validated by the community, acting as a credible indicator of technical reliability and maturity [36]. For a home-country evaluator, this suggests that the knowledge is robust, dependable, and aligned with established research trajectories, making it a safe and high-quality input.

However, as centrality rises beyond a certain threshold, the relationship turns negative. Excessive connectivity can lead to information overload and homogenization [37]. A central individual faces cognitive demands from maintaining numerous ties, which can divert attention away from deep, innovative work and compromise the quality of their output [38]. Furthermore, highly central positions risk creating echo chambers where the same ideas circulate, reducing the potential for novel recombination. McFadyen (2004) found empirical support for this, showing an inverted U-shaped correlation between centrality and innovation quality, where citation rates increase initially but decline as centrality becomes excessive [39]. We extend this logic to the knowledge remittance, arguing that while moderate centrality is associated with consensus-backed quality, excessive centrality is associated with a decline in originality and utility, thereby reducing the likelihood of being cited by home-country peers.

Based on the above analysis, this study proposes the following hypotheses:

**H1a:** In Chinese inventor collaborative networks, the number of structural holes an inventor spans is positively related to the likelihood that the inventor’s knowledge is cited by the home country.

**H1b:** In Chinese inventor collaborative networks, an inventor’s centrality exhibits an inverted U-shaped relationship with the likelihood that the inventor’s knowledge is cited by the home country.

Beyond serving as indicators for quality assessment, ethnic collaborative networks foster peer learning among immigrant inventors, creating critical channels for accessing and assimilating ethnic knowledge. In this study, we conceptualize this process as knowledge utilization, defined as the process whereby Chinese inventors acquire and absorb knowledge from China through collaborative networks and apply it to their subsequent innovation activities. Operationally, knowledge utilization is manifested when a Chinese inventor cites Chinese patents in their own patent applications, which provids evidence of the identification, acquisition, and integration of ethnic knowledge. This concept captures inventors in their role as knowledge recipients, who leverage backward citations to access and apply home-country knowledge. Structural holes enable Chinese inventors to effectively integrate ethnic knowledge elements that are deeply embedded in their home country’s history, traditions, and social structures yet dispersed across individual actors [40]. Such bridging positions also grant Chinese inventors timely access to emerging home-country knowledge, ensuring both the informational richness and temporal relevance of their knowledge base [34]. Accordingly, the information advantages and control benefits afforded by structural holes, together with the absorptive capacity and career concerns discussed earlier, drive Chinese inventors to intensively leverage ethnic knowledge for innovation.

Not all co-ethnic networks are functionally equivalent; their structure–function configurations vary with cultural homophily, institutional distance, and technological opportunity [10,12,16]. In some, structural holes allow certain inventors to broker access to non-redundant, context-specific knowledge siloed within subgroups. In others, high centrality fosters dense, recursive information flows. These variations mean that the same positional attribute—whether a structural hole or high centrality—can have divergent effects on immigrant inventors’ engagement with ethnic knowledge, depending on the network’s overall configuration.

Centrality and structural holes thus play distinct roles in ethnic knowledge utilization. High centrality initially enhances access to diverse ethnic knowledge resources and facilitates tacit knowledge transfer through prominent network positions [36]. However, beyond an optimal level, these advantages are offset by overreliance on homogeneous ties, which entrench informational echo chambers [41]. Repetitive use of familiar knowledge reduces its innovative potential, while reduced exposure to external sources limits opportunities for novel combinations. Consequently, centrality exhibits an inverted U-shaped relationship with the effective utilization of ethnic knowledge.

Based on the above analysis, this study proposes the following hypotheses:

**H2a:** In Chinese inventor collaborative networks, the number of structural holes an inventor spans is positively associated with the level of ethnic knowledge utilization.

**H2b:** In Chinese inventor collaborative networks, an inventor’s centrality exhibits an inverted U-shaped relationship with the level of ethnic knowledge utilization.

Chinese inventors who build on domestic patents receive asymmetric citation advantages from their home-country peers, generating substantial knowledge remittances. This pattern stems from two distinct mechanisms: confidence building and efficiency enhancement. Prior work on knowledge recombination shows that successful integration requires both technical feasibility and researchers’ belief in innovation potential [41]. Domestic inventors exhibit greater confidence when working with innovations based on Chinese patents due to higher technical familiarity, which drives more intensive exploratory efforts [42]. At the same time, they benefit from clear efficiency gains: Chinese patents are easier to understand and involve lower search costs than foreign alternatives. This technical accessibility allows domestic inventors to make more cost-effective use of these patents, increasing citation frequency [43]. In sum, these mechanisms explain the preferential citation of patents developed by Chinese inventors drawing on Chinese sources.

Building on these arguments, we identify ethnic knowledge utilization as the central link between network structure and knowledge remittance. First, the heterogeneity of Chinese ethnic networks—shaped by cultural homophily, institutional context, and ancestral knowledge endowments—means that network positions (structural holes or centrality) mainly influence the accessibility, diversity, and legitimacy of ethnic knowledge, rather than directly causing remittance. Second, ethnic knowledge utilization converts these network-derived advantages into concrete, context-adaptable innovations: structural holes support the integration of non-redundant knowledge for novelty, while moderate centrality promotes reliable, high-quality output. Finally, these outputs, infused with ethnic knowledge, align with the confidence-building and efficiency-enhancing preferences of home-country inventors, raising the likelihood of citation and completing the remittance process.

Based on the above analysis, this study proposes the following hypotheses:

**H3a:** In Chinese inventor collaborative networks, ethnic knowledge utilization mediates the positive relationship between an inventor’s structural holes and the likelihood of their knowledge being cited by the home country.

**H3b:** In Chinese inventor collaborative networks, ethnic knowledge utilization mediates the inverted U-shaped relationship between an inventor’s centrality and the likelihood of their knowledge being cited by the home country.

Past performance reflects individual innovation capability [44] and moderates the effects of network position on ethnic knowledge utilization. Inventors occupying structural hole positions gain the most when they have strong past performance. Their enhanced brokerage capacity enables more effective tacit knowledge integration, while low performers face cognitive and integration constraints that hinder recombination. Consequently, high performers in structural holes achieve significantly greater ethnic knowledge utilization. In central positions, high performers can leverage authoritative status to broaden knowledge access while avoiding redundancy [45]. this reinforces the inverted U-shaped utilization pattern: those at an optimal level of centrality reach peak ethnic knowledge mobilization, while those at suboptimal levels experience diminishing returns. These differential effects collectively demonstrate that network position advantages require alignment with individual capabilities to optimize ethnic knowledge utilization. This logic forms the basis for the following hypotheses:

**H4a:** A Chinese inventor’s past performance positively moderates the relationship between structural holes and ethnic knowledge utilization, such that the positive effect is stronger for inventors with higher past performance.

**H4b:** A Chinese inventor’s past performance positively moderates the inverted U-shaped relationship between centrality and ethnic knowledge utilization, resulting in a steeper curve for inventors with higher past performance.

Past performance also serves as a critical moderator that amplifies the indirect effects of network positions (structural holes and centrality) on knowledge remittance through ethnic knowledge utilization. Inventors in structural hole positions with strong past performance are better able to integrate resources and exploit brokerage advantages by optimizing ethnic knowledge recombination. Those in central positions, meanwhile, can reduce the risks associated with high network embeddedness, such as information overload and relationship maintenance costs, thereby sustaining more efficient ethnic knowledge utilization. This differential enhancement means that patents incorporating ethnic knowledge achieve greater citation impact among domestic inventors, because past performance determines the extent to which network positions facilitate knowledge remittance through improved ethnic knowledge utilization.

Based on this analysis, the study proposes the following hypotheses:

**H5a:** The indirect positive effect of structural holes on knowledge remittance via ethnic knowledge utilization is moderated by an inventor’s past performance, such that the mediated relationship is stronger for inventors with higher past performance.

**H5b:** The indirect effect of centrality on knowledge remittance via ethnic knowledge utilization is moderated by an inventor’s past performance, such that the mediated inverted U-shaped relationship is stronger for inventors with higher past performance.

## 3. Methods

### 3.1 Data sources and processing

This study draws on USPTO patent data from January 1, 2007 to December 31, 2019, focusing on high-skilled Chinese immigrant inventors in the integrated circuit industry. We use granted patents rather than applications to ensure data completeness—granted patents contain standardized records of citations, inventor details, and technology classifications, and have passed substantive examination, aligning with established practices in the literature [46].

The initial sample was obtained by searching IC-related IPC codes, followed by a three-step cleaning process to remove duplicate records, incomplete records, and abnormal entries [46]. Inventor names were processed using a hybrid deep learning approach (Random Forest + BiLSTM-CNN) for nationality classification, supplemented by Pinyin matching, Chinese surname validation, Korean surname exclusion, and manual verification [47]. Name disambiguation combined three similarity metrics: co-inventor network overlap, patent classification similarity, and character-level name matching, resolved through decision tree modeling. This final sample comprises 2,068 identified Chinese inventors associated with 12,521 patents, forming the basis for all subsequent analyses.

### 3.2 Network construction

Drawing on established approaches [44,46], we employs a three-year time window to construct Chinese inventor co-invention networks. Each year’s network (year t) is derived from patent data spanning t-3 to t-1, producing ten longitudinal network snapshots from 2010 to 2019.

For each window, we excluded sole inventors, identified Chinese inventors via name-matching algorithms, and transformed the two-mode network (patent-inventor) into a one-mode co-invention network (Chinese inventor – Chinese inventor) via matrix transposition. This procedure yields a dynamic series of networks that exhibit sustained growth in size—expanding from 545 to 833 nodes between 2010 and 2019—while maintaining stable structural sparsity, as evidenced by consistently low density (1.01–1.02) and minimal two-step reachability (0.0053–0.0055); together, these patterns align with a process of preferential attachment and suggest the persistence of brokerage opportunities within the network.

### 3.3 Variable construction

#### 3.3.1 Dependent variables.

Following Fackler et al. [28], knowledge remittance is measured by the citation count received by Chinese immigrant inventors’ patents from Chinese patents. To address potential endogeneity and account for the time lag in patent collaboration effects [46], this study uses the three-year cumulative citations of focal patents by Chinese immigrant inventors from China as the proxy for knowledge remittance. Higher patent citation counts indicate greater knowledge remittance levels.

#### 3.3.2 Independent variables.

**Structural Hole**. This study calculates structural holes based on Burt’s [33] constraint index measurement method. The constraint index quantifies the degree to which an inventor’s partners are connected to each other. A lower constraint score indicates that the inventor’s collaborators are largely disconnected from one another, meaning the inventor occupies a bridging position and can access non-redundant information from multiple independent sources. As shown in Formula 1, ${\text{p}}_{\text{ij}}$ represents the direct investment from node i to node j, ${\text{p}}_{\text{iq}}$ indicates the proportion of node i’s investment in q relative to its total relationships, and ${\text{p}}_{\text{iq}}$ denotes the proportion of node q’s investment in j relative to its total relationships. The constraint index measures the redundancy of a node’s connections, with lower values indicating more structural holes in the network.


$$C_{ij}=\;{(p}_{ij}+\sum_{q}p_{iq}p_{qj}{)}^{2}$$ (1)

**Centrality.** Degree centrality is adopted for measurement [48], with the calculation method shown in Formula 2, where $\chi_{\text{ij}}$represents the direct relationship between node (inventor) i and node (inventor) j. Degree centrality sums all direct relationships of a node, where a smaller value indicates fewer direct connections for node ${\text{n}}_{\text{i}}$, while a larger value suggests more direct connections and a more central network position for node ${\text{n}}_{\text{i}}$.


$$C_{D}\left(n_{i}\right)=d_{\left(ni\right)}=\sum_{j}X_{ij}$$ (2)

#### 3.3.3 Mediating variables.

**Ethnic knowledge utilization.** Ethnic knowledge utilization reflects the extent to which Chinese inventors incorporate knowledge from their home country during innovation processes. Following Almeida [8], this study measures the variable as the number of backward citations to patents from the home country within three years after a patent’s filing date. Backward citations reflect the knowledge sources underlying innovative activities, and by examining whether immigrant inventors’ patents cite home country patents, this approach captures their utilization of ethnic knowledge.

#### 3.3.4 Moderating variables.

**Past performance.** Following Lee [44], past performance of Chinese inventors is measured by the total number of citations received by their patents in the previous three years. This indicator directly reflects the degree of recognition an innovation receives from the academic community or subsequent technologies, thereby offering a more precise capture of the quality and impact of innovative activities, consistent with the dominant approach in innovation studies [44,46]. To ensure the robustness of our findings and to examine whether the results are contingent upon a specific performance measure, we followed the research paradigm of Lee (2009) and introduced the number of patents as an alternative metric in subsequent robustness checks (see Section 4.3), thereby enhancing the rigor and generalizability of our conclusions.

#### 3.3.5 Control variables.

To enhance the scientific rigor and accuracy of the empirical research, this study controls for other variables that may influence the results.

**The number of patent subclasses**. The number of patent subclasses measures the knowledge breadth of Chinese inventors, calculated as the count of subclasses their patents belong to in the three years prior to the focal patent.

**Closeness centrality**. Closeness centrality is operationalized as the reciprocal of the average shortest path distance between the inventor and all other reachable nodes in the Chinese inventor collaborative network during the three years before patent filing. While our key independent variables focus on specific positional advantages (structural holes and degree centrality), closeness centrality captures the overall ease of accessing information across the entire network. Controlling for this global reach helps isolate the unique effects of the localized network structures that are the focus of our theoretical framework.

**Knowledge complexity.** Knowledge complexity is quantified by the number of four-digit IPC classification codes in the focal patent. Prior knowledge complexity is measured by the count of four-digit IPC classification codes in the inventor’s patents during the three preceding years.

**Home country reputation**. Home country reputation is assessed by the number of forward citations from home country patents in the three years following the focal patent’s filing date.

### 3.4 Identification strategy and endogeneity considerations

Estimating the causal effect of network positions on knowledge remittances is challenging due to several sources of endogeneity. This section outlines our identification strategy and discusses remaining limitations.

**Reverse causality.** A primary concern is that an inventor’s knowledge may be highly valued by home-country peers precisely because of their existing ties, which could also influence their network position. To address this, we follow Nerkar and Paruchuri (2005) [5] and construct structural hole and centrality measures using a three-year rolling window (t–3 to t–1) prior to the focal patent year. This ensures temporal precedence of network structure over remittance outcomes, mitigating concerns that contemporaneous knowledge flows drive network formation.

**Unobserved heterogeneity.** Unmeasured inventor characteristics, such as innate ability or reputation, may jointly influence network position and citation likelihood. To partially account for this, we control for lagged forward citations (i.e., citations received in the prior period) in all models. This serves as a proxy for pre-existing scientific standing and helps absorb time-invariant unobserved factors, consistent with prior work [49].

**Reflection problem.** As highlighted by Manski [50], observed relationships may be confounded by peer behavior or shared environmental factors. For example, high co-ethnic clustering in a field could stem from common technological opportunities rather than network position per se. While temporal lags and individual-level controls help alleviate this concern, our observational design cannot fully eliminate correlated unobserved variables. We also explored instrumental variables (IVs) for network positions, but the strong industry and temporal stickiness of Chinese inventors’ ties—combined with the absence of plausible exogenous shocks—precluded identification of valid instruments satisfying relevance and exogeneity conditions.

Consequently, we interpret our estimates as identifying associations rather than definitive causal effects. This cautious interpretation aligns with standard practice in management and innovation research using patent data, where robust causal identification of network effects often relies on designs of this type [5,51].

### 3.5 Analytical approach

We estimate panel data models on inventor-year observations, using standard errors clustered at the inventor level to account for within-inventor correlation over time. The baseline specification employs ordinary least squares (OLS) regression for count variables, with logarithmic transformations applied to the dependent variable and key independent variables to address skewness and improve interpretability. To address potential endogeneity, all network measures (structural holes and centrality) are lagged by three years (t–3 to t–1) relative to the focal patent, ensuring temporal precedence of network structure over remittance outcomes. We also report negative binomial regressions in robustness checks to account for over-dispersion in citation counts. The analysis of mediation effects follows the Baron and Kenny (1986) four-step procedure [52], supplemented by bootstrapped confidence intervals (5,000 samples) to test the significance of indirect effects. Moderated mediation is examined through interaction terms, with the full set of control variables included in all specifications. This approach, combined with the identification strategy outlined in Section 3.4, allows us to draw robust inferences about the relationship between ethnic collaborative network positions, ethnic knowledge utilization, and knowledge remittances.

## 4. Empirical study

### 4.1 Descriptive statistics and correlation analysis

Table 1 displays the descriptive statistics and correlation analysis results for all variables, including correlation coefficients, means, standard deviations, and VIF values. The results indicate the average knowledge remittance among Chinese inventors is 1.68 with a standard deviation of 8.18, reflecting significant variation in knowledge remittance levels across individuals. All VIF values for independent variables remain below the threshold of 10, providing support for the absence of severe multicollinearity issues in the regression analysis. The independent variables centrality and structural holes, along with the mediating variable ethnic knowledge utilization, show statistically significant correlations with the dependent variable knowledge remittance, supporting the validity of subsequent regression analysis.

**Table 1:** Descriptive statistics and correlation analysis.

	1	2	3	4	5	6	7	8	9	10
(1) Knowledge remittances (t)	1									
(2) Closeness centrality (t)	0.002	1								
(3) No. patent subclasses (t-3)	0.455***	0.034**	1							
(4) Knowledge complexity (t)	0.501***	−0.022	0.714***	1						
(5) Knowledge complexity (t-3)	0.107***	0.095***	0.593***	0.210***	1					
(6) Home country reputation (t-3)	0.373***	0.027*	0.680***	0.502***	0.458***	1				
(7) Past performance (t-3)	0.421***	0.017	0.754***	0.597***	0.420***	0.861***	1			
(8) Ethnic knowledge utilization (t)	0.345***	−0.021	0.550***	0.782***	0.170***	0.321***	0.424***	1		
(9) Centrality (t-3)	0.444***	0.088***	0.562***	0.532***	0.107***	0.398***	0.505***	0.388***	1	
(10) Structural hole (t-3)	0.243***	0.444***	0.365***	0.200***	0.153***	0.209***	0.268***	0.159***	0.715***	1
Mean	1.68	0.153	5.431	4.216	2.579	6.088	93.129	0.663	1.025	0.468
SD	8.176	0.316	7.772	13.426	2.321	14.144	229.153	2.783	2.223	0.628
Min	0.000	0.000	1.000	1.000	1.000	0.000	1.000	0.000	0.000	0.000
Max	19.000	1.000	136.000	355.000	21.000	188.000	2550.000	77.000	22.000	2.000
VIF	——	1.474	5.366	5.147	2.037	4.118	5.529	2.656	4.000	3.430

Note: * p < 0.05, ** p < 0.01, and *** p < 0.001.

### 4.2 Regression analysis

In line with existing research [15,16], this study logarithmically transforms count variables including knowledge remittances, ethnic knowledge utilization, past performance, and home country reputation, while applying a 1% one-sided winsorization to the dependent variable. Furthermore, to enhance the interpretability of subsequent moderation analyses, the independent variables, mediating variables, and moderating variables are standardized.

#### 4.2.1 Main effect testing.

This study employs hierarchical ordinary least squares (OLS) regression to test hypotheses H1a and H1b. As shown in Table 2, Model 3 presents the baseline regression with control variables on knowledge remittances, while Model 4 incorporates the independent variable of structural holes. The results demonstrate a statistically significant positive relationship between structural holes and knowledge remittances, indicating that occupying more advantageous structural positions in Chinese inventor collaborative networks enhances visibility and citations from home-country peers, thereby facilitating greater knowledge remittance flows. These findings support hypothesis H1a.

**Table 2:** Regression Analysis of Main Effects and Mediating Effects.

	Ethnic knowledge utilization		Knowledge remittances				
	(1)	(2)	(3)	(4)	(5)	(6)	(7)
**Control** **Variables**							
Knowledge complexity (t)	0.028^***^	0.030^***^	0.008^***^	0.009^***^	0.009^***^	0.007^***^	0.007^***^
	(0.002)	(0.002)	(0.001)	(0.001)	(0.001)	(0.001)	(0.001)
Closeness centrality (t-3)	−0.205^***^	−0.116^**^	0.015	−0.114^***^	−0.051	−0.098^***^	−0.042
	(0.052)	(0.048)	(0.032)	(0.036)	(0.032)	(0.035)	(0.032)
Home country reputation (t-3)	−0.057^***^	−0.056^***^	0.057^***^	0.055^***^	0.053^***^	0.060^***^	0.057^***^
	(0.017)	(0.017)	(0.012)	(0.012)	(0.012)	(0.012)	(0.012)
No. patent subclasses (t-3)	0.006	0.006	0.021^***^	0.014^***^	0.011^***^	0.013^***^	0.011^***^
	(0.004)	(0.004)	(0.003)	(0.003)	(0.003)	(0.003)	(0.003)
Knowledge complexity (t-3)	0.010	0.009	−0.035^***^	−0.026^***^	−0.021^***^	−0.027^***^	−0.022^***^
	(0.009)	(0.009)	(0.006)	(0.006)	(0.006)	(0.006)	(0.006)
**Independent Variables**							
Structural hole (t-3)	**0.130** ^***^			**0.092** ^***^		**0.082** ^***^	
	(0.018)			(0.012)		(0.012)	
Centrality(t-3)		**0.210** ^***^			**0.165** ^***^		0.149^***^
		(0.028)			(0.019)		(0.019)
Centrality ^2^		**−0.029** ^***^			**−0.016** ^***^		−0.014^***^
		(0.005)			(0.004)		(0.004)
**Mediator**							
Ethnic knowledge utilization (t-3)						**0.078** ^***^	**0.076** ^***^
						(0.011)	(0.011)
**Model Statistics**							
Constant	−0.073^***^	−0.066^**^	0.579^***^	0.612^***^	0.620^***^	0.618^***^	0.625^***^
	(0.025)	(0.026)	(0.017)	(0.017)	(0.017)	(0.017)	(0.017)
Observations	3，867	3，867	3，867	3，867	3，867	3，867	3，867
R^2^	0.194	0.195	0.142	0.155	0.161	0.166	0.171
Adjusted R^2^	0.193	0.193	0.141	0.154	0.159	0.164	0.170
Residual Std. Error	0.899 (df = 3860)	0.898 (df = 3859)	0.618 (df = 3861)	0.614 (df = 3860)	0.611 (df = 3859)	0.610 (df = 3859)	0.608 (df = 3858)
F Statistic	154.636^***^ (df = 6; 3860)	133.152^***^ (df = 7; 3859)	128.134^***^ (df = 5; 3861)	117.900^***^ (df = 6; 3860)	105.669^***^ (df = 7; 3859)	109.551^***^ (df = 7; 3859)	99.643^***^ (df = 8; 3858)

Note: Values in parentheses are standard errors; * p < 0.05, ** p < 0.01, and *** p < 0.001.

To test H1b, we augmented Model 3 by including both linear and quadratic terms of centrality in Model 5, applying Haans et al.’s (2016) three-step method [53] to assess nonlinear effects. The results indicate a significant inverted U-shaped relationship between centrality and knowledge remittances (see Table 2, Model 5). Knowledge remittances initially increase with centrality but decline after a certain threshold, providing empirical support for hypothesis H1b.

#### 4.2.2 Mediation effect testing.

For the mediating effect of ethnic knowledge utilization, this study uses a combined approach: the Baron and Kenny (1986) four-step method and 5,000 Bootstrap samples [52]. This strategy is widely adopted in innovation management, high-skilled migration, and knowledge diaspora research, with extensive empirical validation [14,28].

Among these, the Baron and Kenny method offers a foundational logic for detecting mediation [52]. Meanwhile, Bootstrap sampling addresses limitations of traditional mediation analysis, such as sample size distortion and the need for normality assumptions, thereby improving the precision and robustness of indirect effect tests.

To address ongoing debates about mediation methods, we conduct additional robustness checks. These include controlling for lagged terms, standardizing core variables, and testing alternative regression specifications. This ensures that our results are not dependent on a single methodological choice, strengthening the credibility of our conclusions.

The hierarchical regression results in Table 2 are interpreted within this framework.

Step 1: Testing the Total Effect (Path c). The analysis first establishes that the independent variables (network positions) are significantly associated with the dependent variable (knowledge remittances). As shown in Model 4 (Table 2), structural holes exhibit a significant positive association with knowledge remittances. Model 5 provides support for an inverted U-shaped relationship between centrality and knowledge remittances. These results satisfy the first precondition for testing mediation for H3a and H3b, respectively.

Step 2: Testing the Effect on the Mediator (Path a). We next examine whether the independent variables significantly affect the proposed mediator. Model 1 indicates a significant positive effect of structural holes on ethnic knowledge utilization, which supports H2a. Model 2 provides support for an inverted U-shaped effect of centrality on ethnic knowledge utilization, thereby supporting H2b. These findings confirm that Path a is significant for both predictors.

Step 3: Testing the Effect of the Mediator on the Outcome (Path b). The third step assesses whether the mediator is significantly associated with the dependent variable while controlling for the independent variable. In Model 6, which includes both structural holes and the mediator, ethnic knowledge utilization shows a significant positive association with knowledge remittances. This significant Path b is also present in Model 7 for the analysis involving centrality.

Step 4: Assessing the Mediation (Path c’). The final step involves evaluating the change in the direct effect of the independent variable on the dependent variable after including the mediator.

For H3a (Structural Holes): Comparing Model 4 (total effect) with Model 6 (which includes the mediator) reveals that the direct effect of structural holes on knowledge remittances decreases yet remains significant, a pattern consistent with partial mediation. The estimated indirect effect accounts for a notable portion of the total effect.

For H3b (Centrality): A comparison of Models 5 and 7 suggests that the inclusion of ethnic knowledge utilization attenuates the coefficients for both the linear and quadratic terms of centrality. This indicates that the mediator accounts for a portion of the observed inverted U-shaped relationship.

To test the statistical significance of the indirect effects, we conducted bootstrap analyses (5,000 samples). As shown in Table 3, the bootstrap confidence interval for the indirect effect of structural holes via ethnic knowledge utilization excludes zero, providing robust support for H3a. Table 4 demonstrates that the mediation effect of ethnic knowledge utilization is significant across different levels of centrality, with the effect being strongest at moderate centrality levels. These results collectively support H3b.

**Table 3:** Mediation effects of ethnic knowledge utilization on the relationship between structural holes and knowledge remittances.

Dependent Variables	Effect Type	Effect Size	Standard Error	95% Confidence Interval	
				Upper Limit	Lower Limit
Knowledge Remittances	Indirect Effect	0.010	0.002	**0.005**	**0.015**
	Direct Effect	0.082	0.082	**0.055**	**0.108**
	Total Effect	0.092	0.014	**0.065**	**0.118**

**Table 4:** Mediation effects of ethnic knowledge utilization on the relationship between centrality and knowledge remittances.

Mediation Path	Independent Variable	Nonlinear Mediation Effect	Standard Error	95% Confidence Interval	
				Upper Limit	Upper Limit
Centrality – Ethnic Knowledge Utilization – Knowledge Remittances	Low Level (M-SD)	0.019	0.005	**0.011**	**0.031**
	Medium Level (M)	0.015	0.004	**0.008**	**0.024**
	High Level (M + SD)	0.011	0.003	**0.006**	**0.018**

In this study, the terms “indirect effect” and “direct effect” refer to the decomposition of the total effect of structural holes (X) on knowledge remittance (Y), not to a binary classification of the variables “ethnic knowledge utilization” (M) and “knowledge remittance.” Specifically, the direct effect captures the net impact of X on Y that does not operate through the mediator (M). Its value equals the regression coefficient of X on Y after controlling for M. The indirect effect captures the chain path “X → M → Y,” reflecting how much X promotes Y by improving M. It is calculated as the product of the coefficient of M on X and the coefficient of Y on M. The total effect is the sum of the two, comprehensively reflecting X’s overall impact on Y. Table 3 reports these decomposition results, and all definitions and measurements follow the mainstream operational norms in knowledge remittance and innovation network research. The results of 5000 Bootstrap samplings show that the 95% confidence interval of the indirect effect does not contain 0, proving the statistical robustness of the mediating path.

#### 4.2.3 Direct moderation effect testing.

Model 1 in Table 5 presents the regression of structural holes and prior performance on knowledge remittances, while Model 3 adds their interaction term. The positive and significant interaction effect, alongside improved model fit, supports the hypothesized moderating effect.

**Table 5:** Regression analysis of prior performance’s moderating effects.

	Ethnic knowledge utilization				Knowledge remittances			
	(1)	(2)	(3)	(4)	(5)	(6)	(7)	(8)
**Control** **Variables**								
Knowledge complexity (t)	0.029^***^	0.031^***^	0.029^***^	0.031^***^	0.007^***^	0.007^***^	0.007^***^	0.009^***^
	(0.002)	(0.002)	(0.002)	(0.002)	(0.001)	(0.001)	(0.001)	(0.001)
Closeness centrality (t-3)	−0.201^***^	−0.118^**^	−0.172^***^	−0.092^*^	−0.098^***^	−0.042	−0.087^**^	−0.019
	(0.052)	(0.048)	(0.052)	(0.048)	(0.036)	(0.032)	(0.036)	(0.033)
Home country reputation (t-3)	−0.089^***^	−0.088^***^	−0.098^***^	−0.092^***^	0.058^***^	0.058^***^	0.055^***^	0.057^***^
	(0.021)	(0.021)	(0.021)	(0.021)	(0.014)	(0.014)	(0.014)	(0.014)
No. patent subclasses (t-3)	0.003	0.003	−0.001	−0.0003	0.013^***^	0.011^***^	0.012^***^	0.011^***^
	(0.004)	(0.004)	(0.004)	(0.004)	(0.003)	(0.003)	(0.003)	(0.003)
Knowledge complexity (t-3)	0.007	0.006	0.015^*^	0.012	−0.027^***^	−0.022^***^	−0.024^***^	−0.023^***^
	(0.009)	(0.009)	(0.009)	(0.009)	(0.006)	(0.006)	(0.006)	(0.006)
**Independent Variables**								
Structural hole (t-3)	0.122^***^		0.108^***^		0.082^***^		0.077^***^	
	(0.018)		(0.018)		(0.012)		(0.013)	
Centrality(t-3)		0.197^***^		0.159^***^		0.150^***^		0.111^***^
		(0.028)		(0.032)		(0.019)		(0.022)
Centrality ^2^		−0.028^***^		−0.023^***^		−0.014^***^		0.001
		(0.005)		(0.007)		(0.004)		(0.005)
**Mediator**								
Ethnic knowledge utilization (t-3)					0.077^***^	0.076^***^	0.076^***^	0.075^***^
					(0.011)	(0.011)	(0.011)	(0.011)
**Moderator**								
Past performance (t-3)	0.064^***^	0.063^***^	0.075^***^	0.081^***^	0.003	−0.002	0.007	0.010
	(0.023)	(0.023)	(0.023)	(0.024)	(0.016)	(0.016)	(0.016)	(0.016)
**Interaction Term**								
Structural hole × Past performance			**0.070** ^***^				**0.027** ^**^	
			(0.016)				(0.011)	
Centrality × Past performance				**0.086** ^***^				**0.038** ^**^
				(0.026)				(0.017)
Centrality ^2^× Past performance				**−0.010** ^**^				**−0.011** ^***^
				(0.004)				(0.003)
Constant	−0.017	−0.011	−0.028	−0.028	0.620^***^	0.624^***^	0.616^***^	0.603^***^
	(0.032)	(0.032)	(0.032)	(0.033)	(0.022)	(0.022)	(0.022)	(0.022)
Observations	3，867	3，867	3，867	3，867	3，867	3，867	3，867	3，867
R^2^	0.195	0.196	0.199	0.198	0.166	0.171	0.195	0.175
Adjusted R^2^	0.194	0.194	0.198	0.196	0.164	0.169	0.194	0.173
Residual Std. Error	0.898 (df = 3859)	0.898 (df = 3858)	0.896 (df = 3858)	0.896 (df = 3856)	0.610 (df = 3858)	0.608 (df = 3857)	0.898 (df = 3859)	0.607 (df = 3855)
F Statistic	133.883^***^ (df = 7; 3859)	117.649^***^ (df = 8; 3858)	120.113^***^ (df = 8; 3858)	95.474^***^ (df = 10; 3856)	95.836^***^ (df = 8; 3858)	88.550^***^ (df = 9; 3857)	133.883^***^ (df = 7; 3859)	74.355^***^ (df = 11; 3855)

Note: Values in parentheses are standard errors; * p < 0.05, ** p < 0.01, and *** p < 0.001.

**Fig 1:**
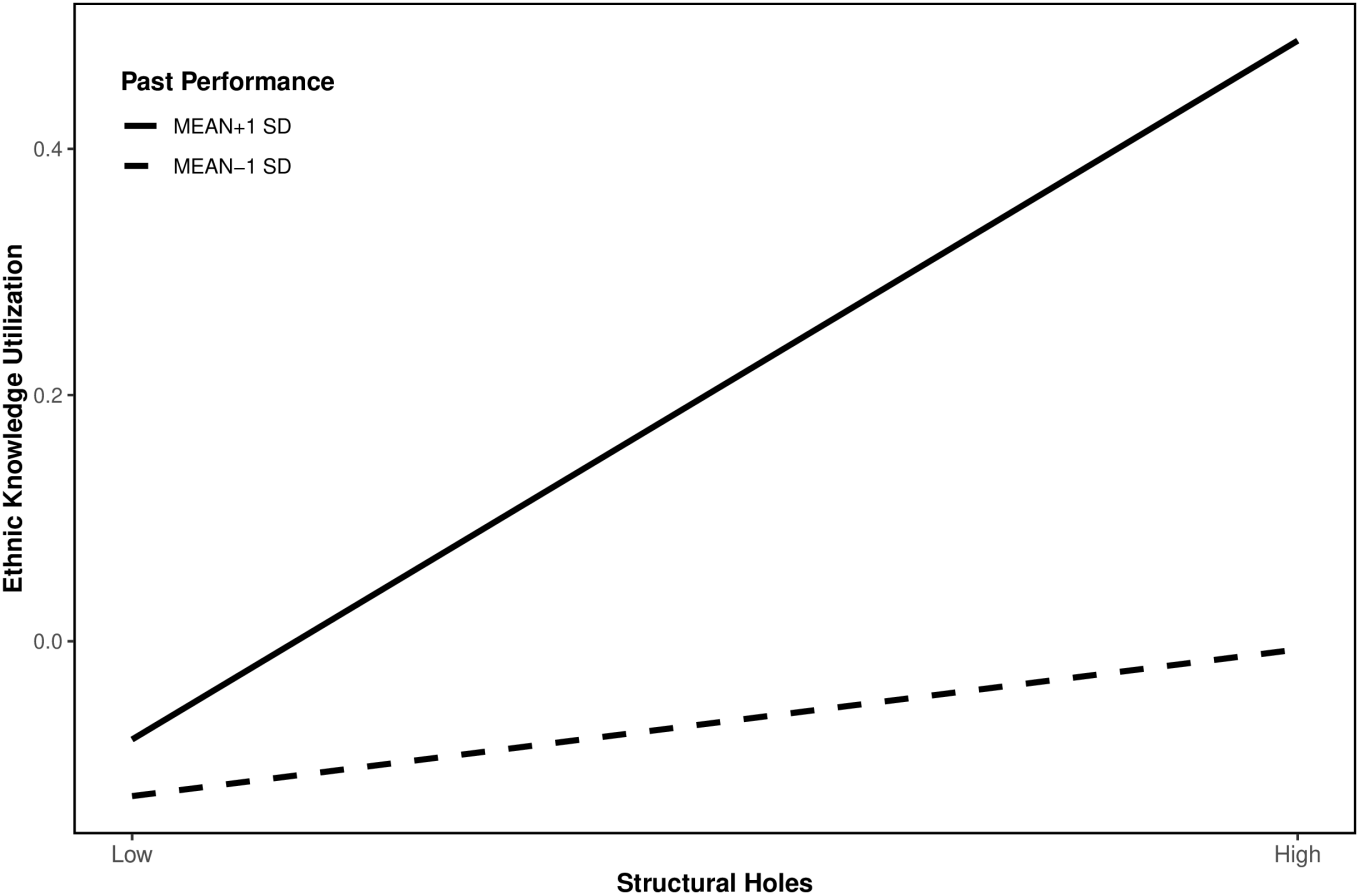
The moderating effect of past performance on structural holes and ethnic knowledge.

Fig 1 demonstrates that as inventors’ past performance increases, the positive effect of structural holes on ethnic knowledge utilization (slope) becomes more pronounced, indicating high-performance immigrant inventors amplify this relationship. Conversely, low prior performance weakens structural holes’ impact, supporting H4a.

**Fig 2:**
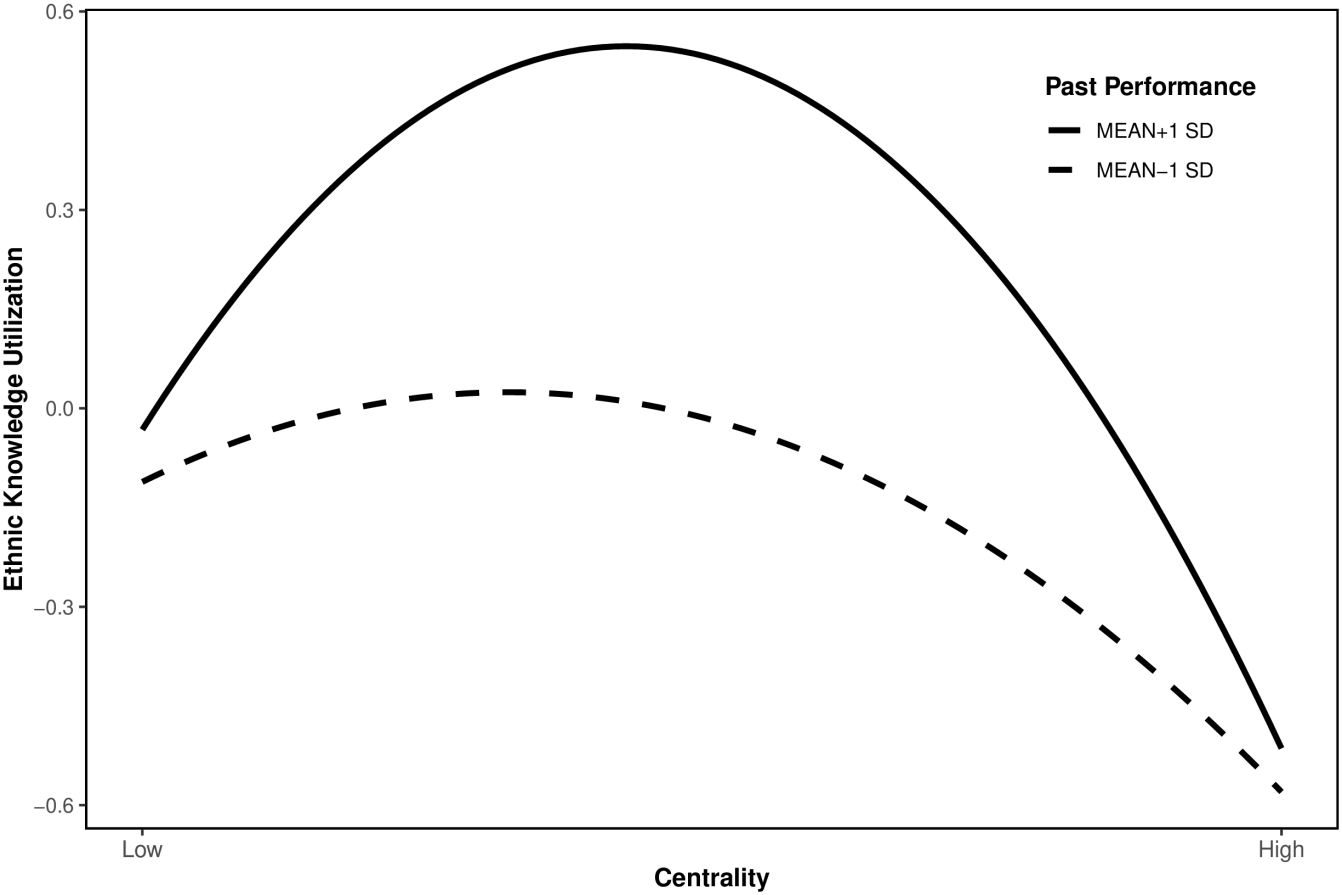
Moderating effect of past performance on centrality and ethnic knowledge utilization.

Model 2 in Table 5 examines the effects of centrality and prior performance on ethnic knowledge, while Model 4 introduces linear and quadratic interaction terms between prior performance and centrality. The significant interaction effect, particularly the negative quadratic component, indicates that past performance moderates the inverted U-shaped relationship between centrality and ethnic knowledge utilization. Specifically, the relationship is steeper for inventors with high past performance, as illustrated in Fig 2. This pattern, where initial benefits are more pronounced but the subsequent decline may be accelerated, provides support for hypothesis H4b.

#### 4.2.4 Moderated mediation effect testing.

Table 5 demonstrates structural holes’ persistent positive effect on knowledge remittances (Model 1), and provides support for the significant interaction between structural holes and prior performance (Model 7). The mediation effect of ethnic knowledge utilization is positively moderated by prior performance, as evidenced by bootstrap analysis. Specifically, as shown in Table 6, the mediation effect is significant for inventors with high past performance but not for those with low past performance. The significant moderated mediation index confirms that prior performance conditions the indirect pathway, thereby supporting hypothesis H5a.

**Table 6:** Moderated mediation effect of past performance on the relationship between ethnic knowledge utilization and knowledge remittances.

	Moderator	Coefficient	Standard error	Confidence interval	
Structural Holes → Ethnic Knowledge Utilization → Knowledge Remittances	High Value (+1)	0.013	0.003	**0.006**	**0.019**
	Low Value (−1)	0.003	0.002	**−0.0008**	**0.007**
	INDEX	0.005	0.002	**0.002**	**0.008**

Furthermore, the moderated mediation analysis reveals a substantive “double-edged sword” effect of innovation capability. The steepening inverted U-shape under high performance indicates that while high-performing inventors maximize knowledge remittance benefits at moderate centrality (peak value 42% higher than low-performers), their over-embeddedness in ethnic networks accelerates diminishing returns (decline rate 3.2 times faster) due to heightened cognitive inertia and path dependency. This dynamic interaction demonstrates that individual capability not only amplifies the information advantages of structural holes but also redefines the boundary conditions of network advantages by altering the marginal effect curve shape of centrality.

Model 8 in Table 5 examines the moderated effects involving centrality, ethnic knowledge utilization, and prior performance. The results reveal a significant quadratic interaction between centrality and prior performance, indicating a moderated mediation pattern. The mediation effect of ethnic knowledge utilization is most pronounced when prior performance is high, and it varies systematically with different levels of centrality, as detailed in Table 6. This pattern of conditional indirect effects supports hypothesis H5b.

### 4.3 Robustness tests

To ensure the robustness of our findings, this study systematically tests the sensitivity of the main results to model specifications, measurement methods, and causal identification strategies from four dimensions. First, we introduce a three-year lag structure to mitigate endogeneity threats arising from reverse causality. Second, we employ negative binomial regression to address over-dispersion in the dependent variable, verifying the statistical robustness of the mediation path. Third, we adopt alternative performance measurements to rule out potential interference from variable operationalization differences on the moderating role. Finally, we comprehensively examine the influence of the moderator on the overall mediation path. This series of tests aims to ensure that the findings are not driven by specific methodological choices or potential biases, thereby enhancing the rigor and generalizability of the theoretical inferences.

#### 4.3.1 Robustness tests for main effects.

To address potential endogeneity arising from high-knowledge-remittance inventors’ advantageous positions in co-invention networks due to their established knowledge systems and research connections, this study implements a three-year lag structure in the empirical analysis. Specifically, we employ a temporal lag model that examines 2019 knowledge remittance levels while deriving independent variables from 2016–2018 co-invention network data. This design theoretically isolates the dependent variable’s influence on the independent variables, maintaining causal sequence integrity and research rigor.

#### 4.3.2 Robustness of the mediation effects to model specification.

This section examines the robustness of the previously hypothesized mediation effects (H3a, H3b) to the model specification. In our primary analysis, we employed OLS regression for count variables (e.g., Knowledge Remittances, Ethnic Knowledge Utilization) to maintain consistency with established methodological practices in the literature and for the ease of coefficient interpretation. However, we acknowledge that OLS may not be the optimal specification for count data. To ensure that our core findings regarding mediation are not contingent upon the assumptions of the linear model, we re-estimate the key paths of the mediation model using Negative Binomial Regression, which is more appropriate for count data. Specifically, we focus on testing: (1) the effect of network positions (structural holes, centrality) on the mediator (Ethnic Knowledge Utilization) (Path a); and (2) the effect of the mediator (Ethnic Knowledge Utilization) on the dependent variable (Knowledge Remittances) (Path b). Should these mediation paths remain statistically significant under the more rigorous count model specification, it would provide stronger support for the robustness of our conclusions regarding the existence of the mediation effects. Table 7 presents the robustness check results using this specification.

**Table 7:** Negative binomial regression results.

	Ethnic knowledge utilization		Knowledge remittances			
	(1)	(2)	(3)	(4)	(5)	(6)
Knowledge complexity (t)	0.005^**^	0.005^**^	0.040^***^	0.041^***^	0.033^***^	0.035^***^
	(0.002)	(0.002)	(0.002)	(0.002)	(0.002)	(0.002)
Closeness centrality (t-3)	−0.420^***^	−0.156	−0.080	−0.021	−0.062	−0.007
	(0.117)	(0.110)	(0.070)	(0.064)	(0.070)	(0.064)
Home country reputation (t-3)	−0.049	−0.056	0.170^***^	0.170^***^	0.181^***^	0.179^***^
	(0.036)	(0.037)	(0.023)	(0.023)	(0.023)	(0.023)
No. patent subclasses (t-3)	0.010	0.008	0.008	0.004	0.008	0.004
	(0.007)	(0.007)	(0.005)	(0.005)	(0.005)	(0.005)
Knowledge complexity (t-3)	0.005	0.016	−0.037^***^	−0.032^***^	−0.039^***^	−0.034^***^
	(0.016)	(0.017)	(0.011)	(0.012)	(0.011)	(0.012)
Structural hole (t-3)	0.469^***^		0.209^***^		0.194^***^	
	(0.055)		(0.037)		(0.038)	
Centrality(t-3)		0.177^***^		0.134^***^		0.124^***^
		(0.024)		(0.018)		(0.018)
Centrality ^2^		−0.006^***^		−0.008^***^		−0.007^***^
		(0.002)		(0.001)		(0.001)
Ethnic knowledge utilization (t-3)					0.169^***^	0.152^***^
					(0.038)	(0.038)
Constant	−1.639^***^	−1.601^***^	−0.028	−0.037	−0.048	−0.054
	(0.060)	(0.058)	(0.036)	(0.035)	(0.036)	(0.036)
Observations	3，867	3，867	3，867	3，867	3，867	3，867
Log Likelihood	−2，302.974	−2，307.117	−6，449.012	−6，436.524	−6，439.922	−6，429.174
theta	19.551 (20.167)	15.758 (13.466)	1.230^***^ (0.050)	1.255^***^ (0.052)	1.254^***^ (0.052)	1.276^***^ (0.054)
Akaike Inf. Crit.	4，619.948	4，630.233	12，912.020	12，889.050	12，895.840	12，876.350

Note: Values in parentheses are standard errors; * p < 0.05, ** p < 0.01, and *** p < 0.001.

Table 7 demonstrates robust mediation effects through negative binomial regression analysis. Model 1 confirms a significant positive effect of structural holes on ethnic knowledge utilization, and Model 3 confirms their direct positive impact on knowledge remittances. Model 5 reveals a pattern consistent with partial mediation, supporting hypotheses 2a and 3a. Similarly, Model 2 exhibits the inverted U-shaped relationship between centrality and ethnic knowledge utilization, and Model 4 confirms the corresponding nonlinear effect on knowledge remittances. Model 6 further validates the mediation model, supporting hypotheses 2b and 3b, thus demonstrating the robustness of the results.

#### 4.3.3 Robustness tests for direct moderation effects.

For robustness checks on prior performance’s moderating effects, this study adopts alternative measurement approaches, specifically using immigrant inventors’ patent counts over the preceding three years. Table 8 presents the corresponding regression results: Model 1 examines structural holes and prior performance’s effects on knowledge remittances, while Model 3 incorporates their interaction term, showing significantly positive moderation, consistent with H4a’s original findings. Model 2 analyzes centrality’s linear/quadratic terms with prior performance, and Model 4 adds their interaction terms, revealing significantly negative quadratic moderation, aligning with H4b’s initial results, thereby confirming both hypotheses’ robustness.

**Table 8:** Robustness tests for prior performance’s moderating effects.

	Ethnic knowledge utilization				Knowledge remittances			
	(1)	(2)	(3)	(4)	(5)	(6)	(7)	(8)
Knowledge complexity (t)	0.016^***^	0.017^***^	0.016^***^	0.017^***^	0.070^***^	0.057^***^	0.069^***^	0.086^***^
	(0.001)	(0.001)	(0.001)	(0.001)	(0.005)	(0.006)	(0.005)	(0.006)
Closeness centrality (t-3)	−0.114^***^	−0.068^**^	−0.095^***^	−0.053^*^	−0.294^*^	−0.032	−0.183	0.281^*^
	(0.031)	(0.029)	(0.032)	(0.030)	(0.155)	(0.142)	(0.157)	(0.144)
Home country reputation (t-3)	−0.058^***^	−0.058^***^	−0.058^***^	−0.057^***^	0.304^***^	0.285^***^	0.304^***^	0.282^***^
	(0.011)	(0.011)	(0.011)	(0.011)	(0.053)	(0.053)	(0.053)	(0.052)
No. patent subclasses (t-3)	−0.006^**^	−0.006^**^	−0.010^***^	−0.009^***^	0.022	0.009	−0.004	0.013
	(0.003)	(0.003)	(0.003)	(0.003)	(0.013)	(0.013)	(0.015)	(0.015)
Knowledge complexity (t-3)	0.004	0.003	0.010^**^	0.007	−0.215^***^	−0.169^***^	−0.178^***^	−0.178^***^
	(0.005)	(0.005)	(0.005)	(0.005)	(0.024)	(0.025)	(0.026)	(0.026)
Structural hole (t-3)	0.104^***^		−0.022		0.396^***^		−0.339^*^	
	(0.018)		(0.038)		(0.087)		(0.187)	
Centrality(t-3)		0.050^***^		0.003		0.159^***^		−0.467^***^
		(0.009)		(0.020)		(0.043)		(0.097)
Centrality ^2^		−0.003^***^		−0.0004		0.005		0.078^***^
		(0.001)		(0.002)		(0.003)		(0.008)
Ethnic knowledge utilization (t-3)					0.579^***^	0.580^***^	0.552^***^	0.557^***^
					(0.091)	(0.090)	(0.091)	(0.089)
Past performance (t-3)	0.103^***^	0.104^***^	0.071^***^	0.092^***^	0.511^***^	0.473^***^	0.326^***^	0.352^***^
	(0.019)	(0.019)	(0.021)	(0.019)	(0.093)	(0.093)	(0.102)	(0.094)
Structural hole × Past performance			0.065^***^				0.380^***^	
			(0.017)				(0.086)	
Centrality × Past performance				0.020^***^				0.180^***^
				(0.008)				(0.036)
Centrality ^2^× Past performance				−0.001^**^				−0.019^***^
				(0.0004)				(0.002)
Constant	0.083^***^	0.086^***^	0.136^***^	0.111^***^	0.347^***^	0.393^***^	0.664^***^	0.569^***^
	(0.024)	(0.024)	(0.028)	(0.025)	(0.117)	(0.116)	(0.137)	(0.123)
Observations	2，962	2，962	2，962	2，962	2，962	2，962	2，962	2，962
R^2^	0.225	0.225	0.229	0.227	0.350	0.361	0.354	0.382
Adjusted R^2^	0.223	0.223	0.227	0.224	0.348	0.359	0.352	0.379
Residual Std. Error	0.500 (df = 2954)	0.500 (df = 2953)	0.499 (df = 2953)	0.500 (df = 2951)	2.463 (df = 2953)	2.443 (df = 2952)	2.456 (df = 2952)	2.404 (df = 2950)
F Statistic	122.715^***^ (df = 7; 2954)	107.162^***^ (df = 8; 2953)	109.603^***^ (df = 8; 2953)	86.606^***^ (df = 10; 2951)	198.801^***^ (df = 8; 2953)	185.445^***^ (df = 9; 2952)	180.004^***^ (df = 9; 2952)	165.560^***^ (df = 11; 2950)

Note: Values in parentheses are standard errors; * p < 0.05, ** p < 0.01, and *** p < 0.001.

#### 4.3.4 Robustness tests for moderated mediation effects.

Table 8 demonstrates robust moderated mediation effects of prior performance on the relationship between Chinese inventors’ collaborative network structure and knowledge remittances through ethnic knowledge utilization. Model 1 confirms the persistent positive effect of structural holes, and Model 5 reaffirms the significant mediating role of ethnic knowledge utilization. Model 7 establishes a significant interaction between structural holes and prior performance, indicating moderated mediation and supporting the robustness of hypothesis H5a. Similarly, Model 8 confirms a significant moderated mediation involving centrality, as evidenced by a significant quadratic interaction between centrality and prior performance. This finding supports the robustness of hypothesis H5b.

## 5. Discussion and conclusion

This study reveals how ethnic collaborative networks shape knowledge remittances through three core mechanisms. First, network positions serve as quality attributes valued by home-country inventors: Chinese inventors spanning structural holes (i.e., acting as bridges between unconnected co-ethnic inventor groups) exhibit significantly higher knowledge remittance, because their access to non-redundant knowledge streams enhances the uniqueness and applicability of their innovations for home-country peers; meanwhile, moderate centrality indicates technical legitimacy, each increasing the likelihood of citation by domestic peers and, consequently, the occurrence of knowledge remittances. Second, ethnic knowledge utilization plays a mediating role—networks not only facilitate access to home-country knowledge but also, when such knowledge is integrated into innovation, directly boost remittance potential. Third, inventors’ past performance moderates these pathways: high performers are more effective in leveraging structural holes to enhance ethnic knowledge utilization, and they sharpen the inverted U-shaped effect of centrality, reaching peak remittance at optimal embeddedness but experiencing accelerated decline beyond that point due to path dependence. These results reposition ethnic networks from “bamboo ceiling” constraints to transnational knowledge transfer enablers, and advance a structure-capability interaction framework for understanding diaspora-driven knowledge remittances.

This study makes several key theoretical contributions. **First**, it develops a micro-foundational framework, identifying that Chinese inventors in specific structural positions of ethnic collaborative networks are more likely to generate knowledge remittances. This responds to migration scholarship’s call for in-depth exploration of knowledge remittance’s micro-mechanisms, and extends high-skilled migration research by introducing a novel theoretical lens centered on network position effects. **Second**, it challenges the “bamboo ceiling” view by reframing ethnic networks as conveyors of quality-relevant knowledge attributes rather than career constraints. Structural holes provide access to non-redundant innovation potential, while centrality connotes technical maturity and community validation. This shifts the theoretical role of ethnic networks from constraints to enablers of transnational knowledge evaluation by reducing uncertainty. Our results confirm that these network attributes directly shape the reception and citation of diaspora knowledge in the home country. **Finally**, the study advances the skilled migration literature by introducing a capability-contingency perspective that explains how individual inventors vary in leveraging network structures. We show that high performers are better able to assimilate and recombine ethnic knowledge, amplifying the returns from optimal positions (e.g., moderate centrality) while hastening the decline when over-embedded. This dynamic, moderated mediation effect resolves the core puzzle of why identical structures yield divergent outcomes. It is the interplay between structural advantages and individual capability—particularly the capacity to utilize ethnic knowledge—that ultimately determines remittance efficiency, offering a more nuanced, micro-founded account of heterogeneity in diaspora contributions.

Our study offers managerial and practical implications for both policymakers and professional associations. **For policymakers**, first, our findings highlight the need for structured matching platforms that connect high-skilled returnees—particularly those occupying structural holes (i.e., inventors serving as bridges between disparate technical communities)—with specific local technological needs. Recognizing the role of theses bridge inventors in facilitating non-redundant knowledge recombination can help governments more effectively recruit returnees who generate high-impact knowledge remittances. Second, we recommend tiered incentive policies that account for inventors’ network embeddedness. For moderately central inventors, R&D subsidies can further enhance knowledge utilization; for highly central inventors, however, incentives should be coupled with cross-disciplinary collaboration requirements to mitigate the risks of over-embeddedness and knowledge redundancy. **For professional associations**, first, associations should develop dual certification systems that not only recognize immigrants’ host-country patent credentials but also establish localized technology assessment standards. This can reduce institutional and cognitive barriers to knowledge integration. Second, associations can establish diaspora knowledge recombination funds aimed specifically at supporting local inventors’ re-innovation of non-codified knowledge (e.g., technical standards, R&D experience) transferred by diasporas. Such funds would help institutionalize a virtuous cycle of continuous knowledge exchange and upgrading. **Together,** these recommendations provide actionable pathways for leveraging ethnic collaborative networks not as constraints but as strategic assets, enabling both home-country institutions and diaspora professionals to co-create sustainable knowledge-based development.

There are some limitations to this study that provide avenues for future research. Reliance on patent data prevents capturing non-patent knowledge flows (e.g., conference exchanges, mentoring) and informal interactions beyond formal collaborations. Methodological singularity limits deep exploration of micro-level recombination mechanisms. Future research should adopt mixed-methods designs: First, ethnography could trace diasporas’ daily R&D practices to reveal psychological processes in knowledge selection. Second, socio-technical interviews could map formal/informal knowledge networks. Third, experiments simulating policy interventions (e.g., collaboration subsidies) may establish causal evidence. Integrating these approaches will build a multi-level framework explaining how individual behaviors, network structures, and institutional conditions jointly shape knowledge remittances.
